# PARP Inhibitors Differentially Regulate Immune Responses in Distinct Genetic Backgrounds of High-Grade Serous Tubo-Ovarian Carcinoma

**DOI:** 10.1158/2767-9764.CRC-24-0515

**Published:** 2025-02-19

**Authors:** Luiza Doro Pereira, Monica Wielgos-Bonvallet, Selim Misirlioglu, Alireza Khodadadi-Jamayran, Petar Jelinic, Douglas A. Levine

**Affiliations:** 1 Division of Gynecologic Oncology, Department of OB/GYN, Laura and Isaac Perlmutter Cancer Center, NYU Langone Health, New York, New York.; 2 Department of Pathology, Applied Bioinformatics Laboratories, NYU School of Medicine, New York, New York.

## Abstract

Immune checkpoint inhibitors (ICI) have revolutionized treatment for several tumor indications without demonstrated benefit for patients with ovarian cancer. To improve the therapeutic ratio of ICIs in patients with ovarian cancer, several different clinical trials are testing combinations with poly(ADP-ribose) polymerase (PARP) inhibitors. Comparing the immunomodulatory effects of clinically advanced PARP inhibitors (PARPi) may help to identify the best partner to combine with ICIs. We examined the treatment effect of talazoparib (a PARP trapper) and veliparib (a solely PARP enzymatic inhibitor) in homologous recombination deficient (HRD) and homologous recombination proficient high-grade serous tubo-ovarian carcinoma (HGSC) cell lines on immune-related gene expression. We discovered and validated that *CXCL8*, *IL-6*, and *TNF* gene expression were upregulated after talazoparib treatment in both OVCAR3 (HRD) and CAOV3 homologous recombination proficient HGSC cell lines. In contrast, veliparib treatment slightly elevated similar genes exclusively in an HRD HGSC cell line model. We expanded these studies to include olaparib, a PARP trapper less potent than talazoparib, and found effects specific to COV361 (*BRCA1* mutant) and OVCAR8 (BRCA1 methylated) HGSC cells but not all HRD HGSC cell lines. Our studies also identified differences among PARP trappers versus veliparib on augmenting *CXCL10* expression. Finally, we show that talazoparib modulates the *CXCL10* response in cGAS-defective cell lines, independent of the cGAS-STING pathway. These mechanistic studies advance our understanding of how different PARPis affect the immune system in various genetic backgrounds.

**Significance:**

This work highlights how different PARPis, especially talazoparib, modulate immune-related gene expression in ovarian cancer cells, independent of the cGAS-STING pathway. These findings may improve our understanding of how different PARPis affect the immune system in various genetic backgrounds.

## Introduction

Immune checkpoint inhibitors (ICI) have transformed cancer therapy but are not yet clinically useful treatments for high-grade serous tubo-ovarian carcinomas (HGSC; ref. 1). HGSCs have low response rates to ICIs due to their immunosuppressive microenvironment (2–4). Combining ICIs with targeted agents that activate or prime the immune system to ultimately improve response rates to immunotherapies are active areas of investigation.

Poly(ADP-ribose) polymerase (PARP) inhibitors are an FDA-approved targeted therapy for patients with HGSCs containing mutations in genes that are essential for homologous recombination double-strand DNA break repair, such as *BRCA1* or *BRCA2*. There are currently five PARP inhibitors (PARPi); veliparib, rucaparib, olaparib, niraparib, and talazoparib, with clinical activity that are either FDA-approved or in clinical trials (5). Two well-accepted mechanisms of these class of agents are inhibition of PARP’s enzymatic activity and PARP trapping (6). Olaparib, rucaparib, niraparib, and talazoparib are classified as both PARP trappers and catalytic inhibitors. Veliparib is defined as mostly a catalytic inhibitor (6, 7).

PARPis also prime the immune system through the upregulation of programmed death-ligand 1 (PD-L1) expression on tumor cells. This targeted therapy has also been shown to stimulate innate immune responses by activating the cGAS-STING cytosolic DNA-sensing pathway and NF-κB transcription factors. The activation of these pathways then leads to the transcription of type 1 interferon (IFN)-regulated genes and pro-inflammatory cytokines, such as *CXCL10*, *CCL5*, *IL-6*, *CXCL8*, or *TNF-α* (8–10)*.* The responses on the cGAS-STING pathway have been shown to be enhanced in *BRCA*-mutant tumors treated with olaparib and rucaparib compared with *BRCA* wild-type tumors (9–11). On the other hand, the two most potent PARP trappers (niraparib and talazoparib) activate the cGAS-STING pathway in both *BRCA* wild-type and deficient models (8–12). However, veliparib’s effect on the activation of this signaling pathway has not been investigated in ovarian cancer cells.

Based on this pre-clinical evidence, all five PARPis are being evaluated in combination with various ICIs in patients with ovarian cancer. Several early trials with the combination of PARPis and immunotherapy showed encouraging results (13–16). A phase Ib/II basket trial of avelumab and talazoparib (the most potent PARP trapper) in patients with platinum-sensitive *BRCA* mutants found that 55% of the patients were alive at 18 months, which suggests a progression-free survival rate higher than typically seen with PARPis or PD-1 inhibitors as single agents (14). In addition, other two early trials also demonstrated activity of the combination therapy in subsets of *BRCA* wild-type and heavily treated patients, in whom single agent therapy has a limited efficacy (13–15). Given these results, phase III clinical trials are ongoing with different PARPis (17).

As these combination therapies are being tested in the clinic, it is yet to be determined whether a specific PARPi may have preferential combinatorial activity compared with other available agents. It is also unclear what patient population should be treated with this combination of agents. To help us address these questions, we sought to define the differences exhibited among PARP trappers versus more PARP enzymatic inhibitors on immune response pathways in homologous recombination deficient (HRD) versus homologous recombination proficient (HRP) HGSC cells. The alterations within various types of HRD HGSC cell lines were also examined in this study. Here, we reveal that there are dissimilarities in the activation of immune response pathways by different PARPis in HGSC cells with diverse genetic backgrounds. Finally, we identify that PARPis upregulate the expression of a type I IFN-regulated gene independent of the STING protein in cGAS-defective HGSC cells.

## Materials and Methods

### Cell culture and drug preparation

OVCAR3, OVCAR4, and OVCAR8 cell lines were obtained from the National Cancer Institute (NCI, RRID:CVCL_0465, CVCL_1627, and CVCL_1629). CAOV3 and HEK-293T cells were purchased from American Type Culture Collection (ATCC, RRID:CVCL_0201 and CVCL_0063). The COV362 cell line was purchased Sigma-Aldrich (RRID:CVCL_02420). All cell lines tested negative for mycoplasma with *Mycoplasma* PCR Detection Kit (ABM) prior to use in these experiments. Cell lines were authenticated by short tandem repeat analysis at IDEXX BioAnalytics. The biological sex of all cell lines was female, and cells were cultured for up to 20 passages. HEK-293T, CAOV3, and COV362 cell lines were maintained in Dulbecco’s Modified Eagle Medium (DMEM) with L-glutamine, 4.5 g/L glucose, and sodium pyruvate supplemented with 10% fetal bovine serum (FBS) and penicillin/streptomycin. OVCAR3, OVCAR4, and OVCAR8 cells were cultured in Roswell Park Memorial Institute (RPMI) 1640 medium supplemented with 10% FBS and penicillin/streptomycin. The seven cell lines used in this study were maintained under standard conditions. Veliparib (catalog #S1004), rucaparib (catalog #S1098), olaparib (catalog #S1060), niraparib (catalog #S7625), and talazoparib (catalog #S7048) were purchased from Selleckchem. All five PARPis were reconstituted in dimethyl sulfoxide (DMSO; Fisher; catalog # 317275) at 10 mmol/L and stored at −80°C for no more than 6 months.

### RNA interference

siRNAs were obtained from GE Dharmacon (SMARTpool: ON-TARGETplus siRNA) and contain a pool of four individual siRNAs targeting STING (L-024333-00-0005). Control siRNA was used as a negative control (Dharmacon, D-001810-10-05). CAOV3 and OVCAR3 cells were transfected with 7.5 μL of DharmaFECT per mL of transfection media and 25 nmol/L of siRNA pool. Twenty-four hours later, the cells were re-plated in 6-well plates and then treated with DMSO, 10 μmol/L veliparib, or 2 μmol/L talazoparib the following day. STING knockdown was confirmed by real time PCR analysis after 48 hours of drug treatment.

### Immunoblotting

Cell pellets were lysed in M-PER (Mammalian Protein Extract Reagent; Thermo Fisher Scientific, catalog #78501) buffer with protease and phosphatase inhibitors (Cell Signaling Technology, catalog #5872S). Protein concentrations were determined using the Pierce Coomassie Plus (Bradford) Protein Assay (ThermoFisher Scientific, catalog #23236) and then analyzed via SDS-PAGE as previously described (18). The nitrocellulose membranes were incubated overnight with the following primary antibodies: STING (Cell Signaling Technology, catalog # 13647; dilution 1:1,000, RRID:AB_2732796) and cGAS (Cell Signaling Technology, catalog # 15102; dilution 1:1,000, RRID:AB_2732795). The immunoblots were then incubated with either the goat anti-rabbit IRDye-800CW (LI-COR Biosciences, catalog #926-32211; dilution 1:20,000, RRID:AB_621843) or the goat-anti-mouse IRDye-680RD secondary antibody (LI-COR Biosciences, catalog #926-68070; dilution 1:20,000, RRID:AB_10956588) and visualized on the Odyssey infrared imager. ERK 2 expression levels were evaluated as a loading control (Santa Cruz Biotechnology, catalog # sc-1647; dilution 1:20,000, RRID:AB_627547).

### RNA extraction and quantitative real-time PCR

Total RNA was isolated using the RNeasy mini kit (Qiagen; catalog # 74124). Two micrograms of RNA was reverse transcribed to generate cDNA using the High-Capacity CDNA Reverse Transcription Kit (Applied Biosystems; catalog # 4368813) as per manufacturer’s instructions. PCR was performed on the QuantStudio 3 Real-Time PCR System using the TaqMan Universal PCR Master Mix (ThermoFisher Scientific, catalog #4324020), and the following probes purchased from Applied Biosystems: *CXCL10* (Hs00171042_m1), *IL-6* (Hs00174131_m1), *CXCL8* (Hs00174103_m1), and *TNF* (Hs00174128_m1). Reactions for each sample were run in triplicate and then normalized to *GAPDH* (Hs03929097_g1).

### Sample preparation for RNA sequencing

RNA extractions were quantified using RNA Nano Chips (Cat. #5067-1511) on an Agilent 2100 BioAnalyzer. RNA sequencing (RNA-seq) library preps were constructed with the Illumina TruSeq Stranded mRNA Library Prep kit (Cat #20020595) using 500 ng of total RNA as input, amplified with 10 cycles of final amplification. RNA-Seq libraries were visualized using High Sensitivity DNA ScreenTape (Agilent, Cat. #5067-5584) on the Agilent TapeStation 2200 instrument. Quant-It (Invitrogen, Cat. P11495) was used for final concentration determination, and libraries were pooled equimolar. The pool was sequenced single-end 50 cycles on two lanes of an Illumina HiSeq4000 flowcell with 2% PhiX spike-in.

### RNA-seq data processing

RNA-seq data were analyzed by the sns rna-star pipeline (https://github.com/igordot/sns/blob/master/routes/rna-star.md). Sequencing reads were mapped to the reference genome (hg19) using the STAR aligner (v2.6.1d, RRID:SCR_004463; ref. 19). Alignments were guided by a gene transfer format (GTF) file. The mean read insert sizes and their standard deviations were calculated using Picard tools (v.2.18.20; RRID:SCR_006525; http://broadinstitute.github.io/picard). The read count tables were generated using subread (v1.6.3, RRID:SCR_009803; ref. 20) and normalized based on their library size factors using DEseq2 (21), and differential expression analysis was performed. The read per million–normalized BigWig files were generated using BEDTools (v2.26.0, RRID:SCR_006646; ref. 22) and bedGraphToBigWig tool (v4). To compare the level of similarity among the samples and their replicates, we used two methods: principal-component analysis and Euclidean distance-based sample clustering. All the downstream statistical analyses and generating plots were performed in R environment (v3.5.1; https://www.r-project.org/, RRID:SCR_001905). The results of gene set enrichment analysis (GSEA) were generated by GSEA software (23).

### ELISA

Supernatants were collected 48 hours after PARPi treatment, centrifuged at 1,500 rpm for 10 minutes, and stored at −80°C until use. Secreted IL-8 (catalog #D8000C) and IP-10 (catalog #DIP100) were quantified using Human Quantikine enzyme-linked immunosorbent assay (ELISA) kits from R&D Systems.

### Statistical analysis

Data were analyzed using two-sided *t* tests by GraphPad Prism version 8.3.0 (GraphPad Software; RRID:SCR_002798). Data are presented as average ± standard deviation (SD) and were considered significant if *P* < 0.05.

### Data availability

The data generated in this study are available within the article and its supplementary data files. Additional raw data generated in this study are available upon request from the corresponding author. Raw data for RNA-seq were generated at the NYU Genome Technology Center. RNA-seq data have been deposited in the Gene Expression Omnibus database under the accession code GSE285827.

## Results

### Talazoparib treatment stimulated alterations in gene expression

To assess the immunomodulatory effects of PARP trappers versus solely PARP enzymatic inhibitors in HRD and HRP HGSC cell lines, we performed RNA-seq. For this study, we focused on the CAOV3 and OVCAR3 HGSC cells. The OVCAR3 cell line is an HRD cell line as it contains an EMSY amplification, which we have previously demonstrated represents an HRD phenotype (18, 24), whereas CAOV3 is considered an HRP cell line (25). Both ovarian cancer cells were treated with a vehicle control (DMSO), 10 μmol/L veliparib (non-PARP trapper), or 2 μmol/L talazoparib (most potent PARP trapper) for 48 hours prior to analysis by RNA-seq. We utilized this high dose of talazoparib, based on previously published work by Shen and colleagues (8), in which 2 μmol/L talazoparib was shown to activate antitumor immune responses in ovarian cancer cell lines, independent of BRCA status. Additionally, we selected 10 μmol/L veliparib as it had been reported to be a clinically achievable dose (26).

**Figure 1:**
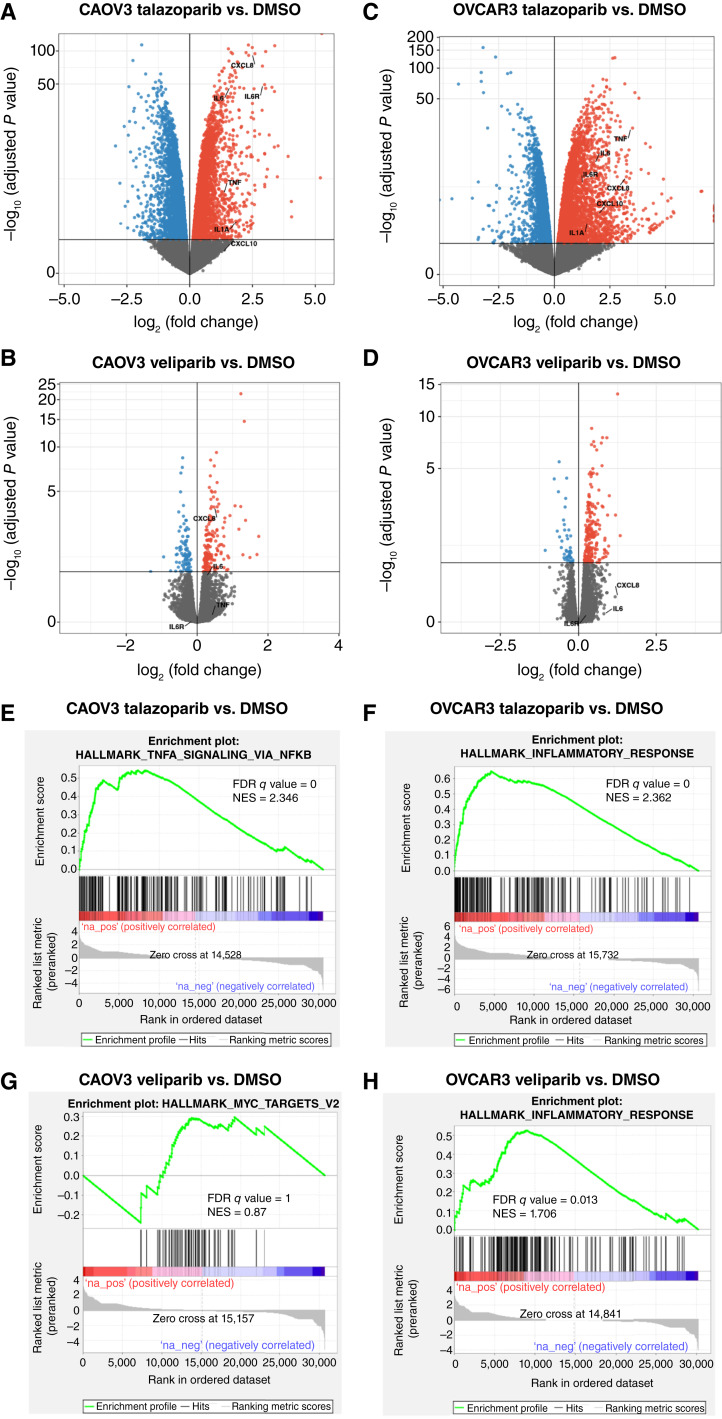
Pro-inflammatory genes are significantly upregulated in CAOV3 and OVCAR3 talazoparib-treated cells compared with control and veliparib-treated cells. Volcano plots highlighting fold change values of a small set of pro-inflammatory genes (FDR < 0.1) in (**A** and **C**) talazoparib-treated vs. DMSO-treated cells or (**B** and **D**) veliparib-treated vs. DMSO-treated cells. Red circles denote genes that were significantly upregulated, and blue circles represent genes that were significantly downregulated. Results shown are from three independent experiments. **E–H,** GSEA plots representative of the top upregulated pathway in each of the four comparisons. NES, normalized enrichment score.

Initially, we measured Euclidean sample-to-sample distances and detected clear clustering among the two cell lines. Clustering of the DMSO- and veliparib-treated cells in both cell lines was also observed to be distinct from talazoparib-treated cells (Supplementary Fig. S1). To identify genes that are differently deregulated between the two PARPis in relationship to DMSO-treated cells, differential gene expression analyses were performed using a 10% false discovery rate (FDR; Fig. 1A–D).

Talazoparib treatment in the CAOV3 cell line elicited the alteration of 7,722 genes compared with 281 genes after veliparib treatment. In OVCAR3 cells, talazoparib deregulated 7,780 genes and veliparib affected the expression levels of 279 genes. This analysis also identified 3,846 genes that were induced in both cell lines following talazoparib treatment (Supplementary Fig. S2A). Treatment with veliparib resulted in a common set of 12 upregulated genes (Supplementary Fig. S2B). These results indicated that the transcriptomes of ovarian tumors endure dynamic modifications following the use of a PARP trapper versus a catalytic inhibitor.

### Immune-related gene expression signatures are upregulated by talazoparib versus veliparib treatment

Next, a GSEA was used to determine the gene expression signatures that were most significantly upregulated in talazoparib- versus veliparib-treated cells (Fig. 1E–H). The top 12 upregulated pathways enriched among both cell lines treated with 2 μmol/L talazoparib included 5 immune-related gene expression signatures, such as the hallmark inflammatory, IL-6 JAK STAT3, TNFA signaling, IFN γ, and IFN α response pathways (Supplementary Tables S1 and S2). Furthermore, the 12 most significantly enriched gene sets in OVCAR3 veliparib-treated cells also included 4 of the 5 immune-related gene expression signatures observed in talazoparib-treated cells, except for the hallmark IL-6 JAK STAT3 response pathway (Supplementary Table S3). The five immune response pathways were neither upregulated nor downregulated in CAOV3 veliparib-treated cells (Supplementary Table S4). Thus, talazoparib activated immune-related pathways in both cell lines. On the other hand, veliparib’s effect on these pathways was specific to the OVCAR3 (HRD) cell line.

### Talazoparib treatment enhanced the gene and protein expression levels of pro-inflammatory cytokines

**Figure 2:**
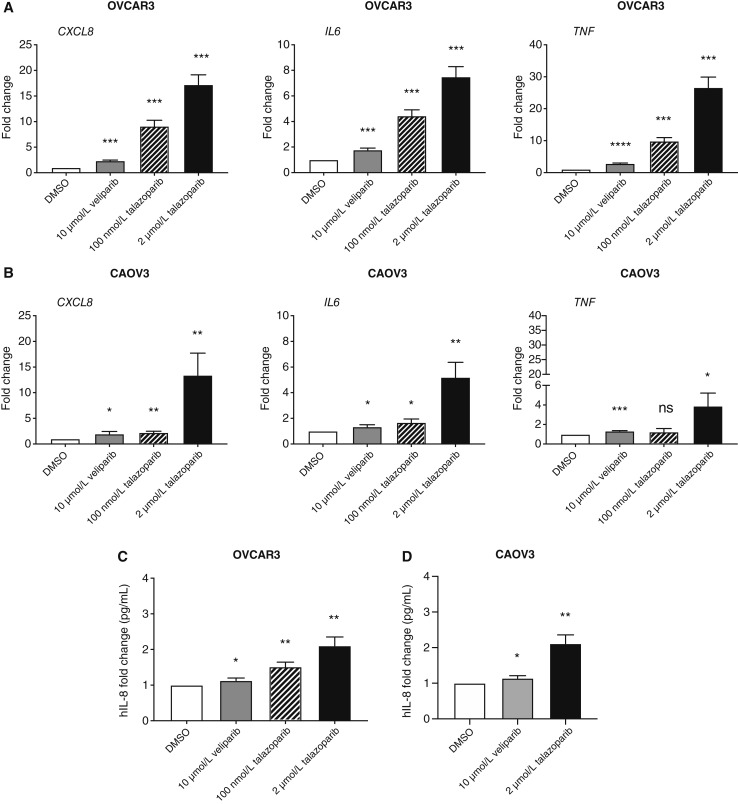
Expression levels of pro-inflammatory genes and proteins are increased by talazoparib treatment in two HGSC cell line models. Total RNAs were isolated from (**A**) OVCAR3 and (**B**) CAOV3 cells treated with DMSO, 10 μmol/L veliparib, 100 nmol/L talazoparib, or 2 μmol/L talazoparib for 48 hours. Two micrograms of isolated RNA was reverse transcribed to cDNA and then analyzed by qRT-PCR for *CXCL8*, *IL-6*, and *TNF* expression. *GAPDH* was used as a housekeeping gene. The supernatants from (**C**) OVCAR3 and (**D**) CAOV3 cells were collected 48 hours after the addition of DMSO, 10 μmol/L veliparib, 100 nmol/L talazoparib, or 2 μmol/L talazoparib. IL-8 protein expression was then analyzed by an ELISA assay. A four-parameter logistic curve fit was used to analyze these results. Means are representative of three independent experiments performed in triplicate in all qRT-PCR and ELISA experiments. *T* tests were performed between DMSO- and the individual PARPi–treated groups. *, *P* < 0.05; **, *P* < 0.01; ***, *P* < 0.001; ns, not statistically significant.

To validate our RNA-seq data, we measured changes in *CXCL8*, *IL-6*, and *TNF* gene expression levels using quantitative RT-PCR analysis. The focus was on these genes as they are involved in the two most commonly upregulated gene sets, the hallmark inflammatory and TNFA signaling response pathways, in CAOV3 and OVCAR3 talazoparib-treated cells as well as in OVCAR3 veliparib-treated cells (Supplementary Tables S1–S4). The mRNA levels of all three genes were significantly upregulated 48 hours after talazoparib treatment in both cell lines (Fig. 2A and B). We also observed a significant, slight increase in expression levels of these three genes in the veliparib-treated groups compared with the DMSO-treated groups (Fig. 2A and B). The fold change values in the OVCAR3 treated cells were greater than the differences observed in the CAOV3 treated cells. These results were very similar to the normalized fold change values we obtained from our RNA-seq experiment.

Additionally, both cell lines were treated with 100 nmol/L talazoparib in order to determine whether a reduced dose would produce similar changes to those exhibited by 2 μmol/L talazoparib. There was a dose dependent response effect observed in the OVCAR3 cell line, and the reduced dose of talazoparib significantly increased expression levels of all three genes but to a lesser extent than 2 μmol/L talazoparib (Fig. 2A). The expression levels were reduced by ∼35% to 60% in the cells treated with 100 nmol/L talazoparib compared with cells treated with 2 μmol/L talazoparib. In the CAOV3 cells, 100 nmol/L talazoparib created more blunted changes in expression than seen with 2 μmol/L talazoparib (Fig. 2B).

Next, we confirmed whether the alterations at the transcript level would be directly translated to protein, using ELISA analysis. Consistent with our qRT-PCR results, 2 μmol/L talazoparib increased IL-8/*CXCL8* secretion in both cells (Fig. 2C and D). The production of IL-8 in OVCAR3 and CAOV3 cells was also slightly altered by 10 μmol/L veliparib. In line with our qRT-PCR results, 100 nmol/L talazoparib increased IL-8 secretion in OVCAR3 cells but not to the same extent as 2 μmol/L talazoparib (Fig. 2C). These data further demonstrated that higher doses of the PARP trapper, talazoparib, activated the production of pro-inflammatory cytokines, independent of HRD status.

### Secretion of pro-inflammatory cytokines by the less potent PARPi, olaparib, is specific to the *BRCA1* mutant and methylated HGSC cells and not all HRD HGSC cells

**Figure 3:**
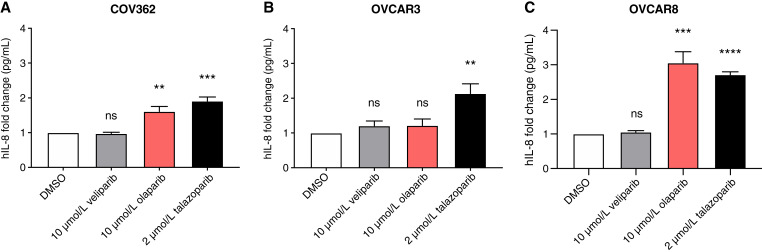
IL-8 *(CXCL8)* is exclusively secreted in BRCA1 methylated or mutated HGSC cells after olaparib treatment but is secreted in all HRD HGSC cells after talazoparib treatment. **A,** OVCAR3, (**B**) OVCAR8, and (**C**) COV362 cells were treated with DMSO, 10 μmol/L veliparib, 10 μmol/L olaparib, or 2 μmol/L talazoparib for 48 hours. The supernatants were then collected, and IL-8 protein expression was analyzed by an ELISA assay. A four-parameter logistic curve fit was used to analyze these results. *T* tests were performed between DMSO- and the individual PARPi–treated groups. **, *P* < 0.01; ***, *P* < 0.001; ****, *P* < 0.0001; ns, not statistically significant.

We next investigated whether a less potent PARP trapper, olaparib, would generate similar changes in the OVCAR3 cell line as those described above after talazoparib treatment. Further, the immunomodulatory effect of olaparib had been primarily assessed in *BRCA*-mutant HGSC cells and not in other HRD tumors. For this reason, we examined the impact of 10 μmol/L olaparib on IL-8 protein expression using an ELISA assay in two HRD HGSC cell lines without a mutation in *BRCA1*: OVCAR3 (EMSY amplified; ref. 18) and OVCAR8 (*BRCA1* promoter methylated; ref. 27). We compared the effects to treatment with 10 μmol/L veliparib and 2 μmol/L talazoparib. The COV362 (*BRCA1* mutant) cell line served as our positive control as olaparib had been previously shown to activate an immune response in this specific genetic background (9, 28). Olaparib and talazoparib both increased IL-8 production in the COV362 cell line, thus confirming previously published data (Fig. 3A). In the OVCAR3 cell line, treatment with 10 μmol/L olaparib slightly increased the production of the IL-8. This effect was similar to that observed after treatment with 10 μmol/L veliparib but less than that seen with 2 μmol/L talazoparib (Fig. 3B). Olaparib treatment elevated IL-8 protein expression to a similar extent as 2 μmol/L talazoparib in the OVCAR8 cell line and more than that with veliparib (Fig. 3C). Taken together, these results demonstrate that PARP trappers differentially regulate pro-inflammatory cytokine secretion in distinct genetic backgrounds.

### Regulation of the type 1 IFN-stimulated gene, *CXCL10*, is PARPi– and cell line–specific

**Figure 4:**
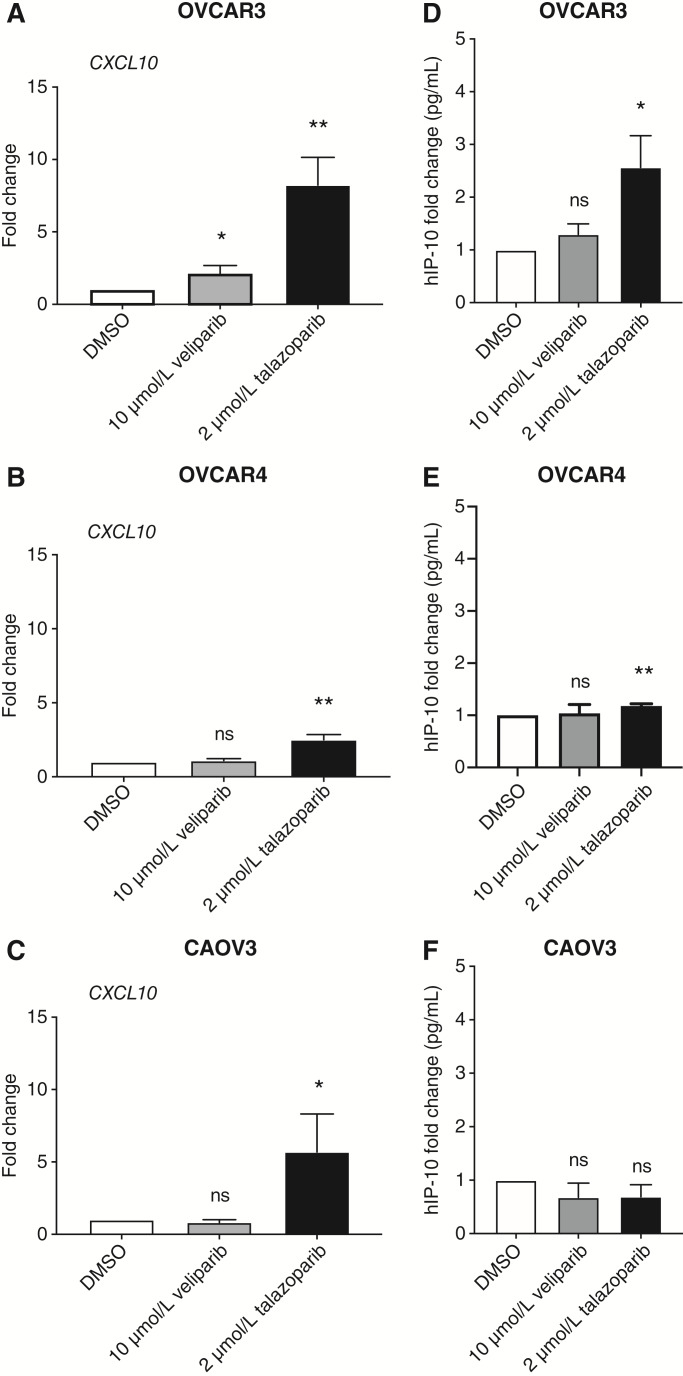
Talazoparib treatment upregulates *CXCL10* gene expression in three, BRCA wild-type HGSC cell lines compared with veliparib treatment. **A** and **D,** OVCAR3, (**B** and **E**) OVCAR4 cells, and (**C** and **F**) CAOV3 cells were treated with a vehicle control (DMSO) or 10 μmol/L Veliparib or 2 μmol/L Talazoparib. Cell pellets and supernatants were collected 48 hours after drug treatment. **A–C,** Two micrograms of total RNA extracted from cell pellets was reverse transcribed to cDNA and then analyzed by qRT-PCR for *CXCL10* and *GAPDH* expression. **D–F,** Supernatants were tested for *CXCL10* (IP-10) protein levels using an ELISA assay. A five-parameter logistic curve fit was used to analyze these results. *, *P* < 0.05; **, *P* < 0.01; ns, not statistically significant.

We continued to define the differences exhibited among PARP trappers versus solely PARP enzymatic inhibitors in relationship to the cGAS-STING innate immunity pathway. This pathway had been previously shown to be activated by the four PARP trappers (29). To assess the activation of the cGAS-STING pathway, we measured the gene and protein expression levels of *CXCL10.* This chemokine is a major target gene downstream of the cGAS-STING pathway that is involved in T-cell chemotaxis. We initially treated three *BRCA* wild-type HGSC cell lines with a vehicle control (DMSO), 10 μmol/L veliparib, or 2 μmol/L talazoparib for 48 hours prior to qRT-PCR or ELISA analysis. The *CXCL10* mRNA levels were significantly elevated by talazoparib treatment compared with veliparib treatment in all three *BRCA* wild-type HGSC cells (Fig. 4A–C). The impact of talazoparib was blunted in OVCAR4 treated cells compared with OVCAR3 and CAOV3 treated cells (Fig. 4B). We also observed a significant, small increase in *CXCL10* expression in the OVCAR3 veliparib-treated cells compared with the OVCAR3 DMSO-treated cells (Fig. 4A). Furthermore, these results were translatable to protein expression in OVCAR3 and OVCAR4 cell lines, in which CXCL10 protein expression was significantly increased with talazoparib treatment, but not veliparib (Fig. 4D and E). IP-10 (*CXCL10*) protein levels were not affected by veliparib and talazoparib treatment in CAOV3 cells (Fig. 4F).

Next, we compared the effects of the five most clinically active PARPis on CXCL10 gene and protein expression in the HRD HGSC cell line, OVCAR3. To assess these differences, we used the same dose of 10 μmol/L for all five agents. We observed that all five PARPis significantly upregulated the mRNA levels of *CXCL10* (Supplementary Fig. S3A)*.* Notably, the greatest effects on this gene were detected after olaparib, niraparib, and talazoparib treatment (Supplementary Fig. S3A). On the other hand, IP-10 protein levels were increased only after olaparib, niraparib, and talazoparib treatment (Supplementary Fig. S3B).

We further investigated the effect of PARP trappers versus solely PARP enzymatic inhibitors in relationship to the cGAS-STING innate immunity pathway in two other HRD cell lines. The *CXCL10* gene and protein levels in the OVCAR8 (*BRCA1* methylated) cell line were upregulated upon treatment with the two PARP trappers, olaparib and talazoparib (Supplementary Fig. S4A and S4B). We also observed a significant, small increase in *CXCL10* mRNA levels in the OVCAR8 veliparib-treated group compared with the OVCAR8 DMSO-treated group (Supplementary Fig. S4A). Similar findings were observed for protein expression in the COV362 (*BRCA1* mutant) cell line (Supplementary Fig. S4C). Combined, our data show that there are differences exhibited among PARP trappers versus solely PARP enzymatic inhibitors on innate immune response pathways in both HRD and HRP HGSC cells.

### *CXCL10* expression induced by PARPis is independent of the STING protein in cGAS-defective cells

**Figure 5:**
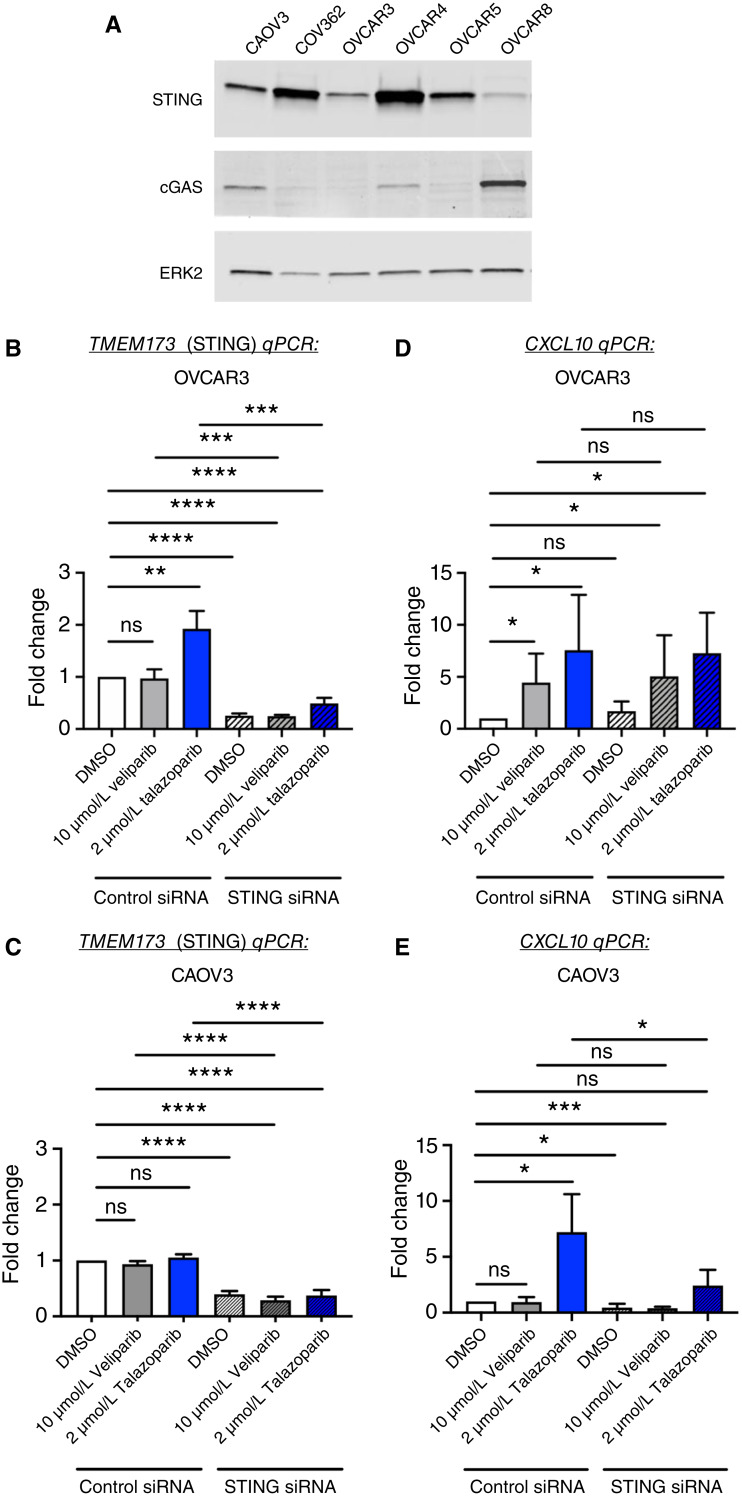
PARPi–induced *CXCL10* gene expression levels are not affected by STING knockdown in a cGAS-defective cell line. **A,** The expression levels of STING and cGAS were measured by western blot analysis 48 hours after DMSO treatment. ERK2 was used as a loading control. Results shown are from one of two independent experiments. **B–E,** OVCAR3 and CAOV3 were transfected with control or STING siRNA for 24 hours. Cells were re-plated the next day and treated 48 hours after transfection with DMSO, 10 μmol/L veliparib, or 2 μmol/L talazoparib. Total RNA was extracted from cell pellets that were collected 48 hours after drug treatment, and then 1 μg of RNA was reverse transcribed to cDNA. Quantitative real-time RT-PCR was utilized to measure the gene expression levels of *TMEM-173* (STING), *CXCL10*, and *GAPDH*. *T* tests were performed between the control siRNA DMSO-treated group and the five other conditions. A *t* test was also performed between the control siRNA veliparib- vs. STING siRNA veliparib-treated groups and the control siRNA talazoparib- vs. STING siRNA talazoparib-treated groups. *, *P* < 0.05; **, *P* < 0.01; ***, *P* < 0.001; ****, *P* < 0.0001; ns, not statistically significant.

The cGAS-STING signaling pathway was previously reported to be defective in a large portion of ovarian cancer cell lines and tumors. To validate these findings, we assessed the cGAS and STING protein expression levels in a panel of HGSC cell lines using western blot analysis. We observed lack of cGAS expression in OVCAR3 cells as reported previously (30). cGAS expression was also absent in the COV362 and OVCAR5 cell lines. Conversely, CAOV3, OVCAR4, and OVCAR8 cell lines expressed both the cGAS and STING protein (Fig. 5A).

As we observed that talazoparib triggered robust CXCL10 expression in cGAS-deficient cell lines (COV362 and OVCAR3), we tested whether STING expression was required to initiate this immune response. To assess whether STING could be inducing *CXCL10* expression, we silenced STING using a pooled *TMEM-173* siRNA in a cGAS-deficient (OVCAR3) as well as in a cGAS-proficient (CAOV3) cell line. CAOV3 also served as positive control as it contained an intact cGAS-STING pathway. We then treated the cells with the vehicle control (DMSO), 10 μmol/L veliparib, or 2 μmol/L talazoparib for 24 hours after transfection. As shown in Fig. 5B and C, *TMEM-173* mRNA levels were reduced by more than 50% in the *TMEM-173* siRNA-treated cells compared with control siRNA-treated cells. Furthermore, *CXCL10* gene expression was upregulated in response to both PARPis in the OVCAR3 control siRNA-treated cells. However, *TMEM-173* (STING) knockdown did not alter *CXCL10* mRNA levels that were elevated by veliparib or talazoparib treatment (Fig. 5D). Conversely, the increase in *CXCL10* expression triggered by talazoparib treatment was decreased by *TMEM-173* (STING) knockdown in the CAOV3 cell line (Fig. 5E). These results suggest OVCAR3 cells can still build an immunogenic response upon DNA damage by a cGAS-STING pathway–independent mechanism.

## Discussion

Recent studies have reported that PARPis have immunomodulatory effects. This class of agents have been shown to recruit T-cells to tumor cells as a result of unrepaired DNA double-strand breaks. The two early trials which performed exploratory analysis suggested that the response to the combination is mediated by an IFN response, which is blunted in non-responders, in accordance with the findings of preclinical studies (9, 13, 15).

However, the importance of PARP catalytic inhibition versus PARP trapping in generating this IFN response is still not well understood. A single preclinical study generated cell lines with a mutant PARP protein, which cannot be trapped in the chromatin, and found those cells have impaired IFN production following talazoparib treatment (29). Here, we demonstrated that the immune gene regulation following PARPi treatment is specific to the PARP trapping potency of the agent and the genetic background of the treated cells. Olaparib, a less potent PARP trapper, was sufficient to trigger IL-8 gene response in the *BRCA1* mutant and methylated cells, but the *EMSY*-amplified cells would only upregulate IL-8 in response to the most potent PARP trapper, talazoparib. Other *in vivo* studies evaluating the immune response to olaparib in ovarian and triple-negative breast cancer showed it to be specific to HRD models. However, those studies examined exclusively *BRCA1*-deficient models, and other types of HRD tumors were excluded (9, 28). Here, we demonstrated the heterogeneity of the response of HRD cancer cells to PARP inhibition. Consistently with the literature, talazoparib could elicit an immune response in all ovarian cancer cells regardless of genetic background and homologous recombination status (8).

PARPis have been reported to elicit a cGAS-STING–dependent immune response in both *BRCA*-mutated and wild-type models (8, 9, 29). However, cGAS-STING is reported to be defective in a large portion of HGSC, and there have been limited efforts to evaluate the immune response to PARPis in cGAS-STING–deficient models of ovarian cancer (30). Pantelidou and colleagues (28) showed that in a cGAS-proficient model of triple-negative breast cancer, silencing of *STING* abrogates *CXCL-10* upregulation following olaparib treatment. These results are concordant with our findings in cGAS-positive CAOV3 talazoparib-treated cells. Based on our findings, the CAOV3 cell line relies on the cGAS-STING pathway to trigger an immunogenic response to PARP trapping (8, 29). On the other hand, *STING* knockdown did not have an effect on talazoparib-induced *CXCL10* expression in a cGAS-deficient cell line, OVCAR3. These results also suggest that defective cGAS-STING signaling may still benefit from the combination of PARPis with immune checkpoint blockade. Elucidating which alternative cytosolic DNA-sensing mechanism is activated after PARP trapping in cGAS-STING–defective tumor cells is an area for further research. It is possible that cGAS-STING–defective tumor cells can stimulate an immune response via cross-activation of cGAS-STING in the dendritic cells (9). Conversely, a phase I/II clinical trial testing the durvalumab and olaparib combination showed minimal increase in both the STING protein and mRNA levels in post-treatment tumor biopsies, yet those samples had increase in *CXCL10* and other immune mediators by RNA-seq (15).

We acknowledge certain limitations of our study, particularly that it was carried out in *in vitro* models and utilized a limited selection of HGSC cell lines. We also limited our investigation of the cGAS-STING pathway to the expression of *CXCL10*, and future studies may expand to include other downstream targets of the cGAS-STING pathway such as type I IFN γ, phospho-TBK1, and phospho-IRF3. Additionally, the absence of *in vivo* models in our study may limit generalizability until further *in vivo* confirmation is performed.

Our findings describe the tumor cell–intrinsic immunogenic activation elicited by different PARPis in a broad range of ovarian cancer cell lines. We demonstrated that the secretion of inflammatory cytokines was PARP– and cell line–specific. We reiterated the relationship between PARP trapping potency and the activation of *CXCL10* response and showed variation among PARPis. One limitation of our study is that we investigated the immune response to PARPis *in vitro* only. Translation of these findings *in vivo* will remain to be determined. Specific PARPis that should be considered in patients with distinct genetic backgrounds will need to be investigated further in preclinical and clinical trials.

## Supplementary Material

Figure S1 Supplementary Figure 1 shows clustering of drug-treated cells compared to controls.

Figure S2 Supplementary Figure 2 shows Venn diagrams of elevated genes in drug-treated cells.

Figure S3 Supplementary Figure 3 shows CXCL10 gene and protein expression levels in drug-treated cell lines.

Figure S4 Supplementary Figure 4 shows IP-10 (CXCL10) expression levels in drug-treated cell lines.

Table S1 Supplementary Table 1 shows enriched gene sets upregulated in CAOV3 cells treated with talazoparib.

Table S2 Supplementary Table 2 shows enriched gene sets upregulated in OVCAR3 cells treated with talazoparib.

Table S3 Supplementary Table 3 shows enriched gene sets upregulated in OVCAR3 cells treated with veliparib .

Table S4 Supplementary Table 4 shows enriched gene sets upregulated in CAOV3 cells treated with veliparib .
