# Correction: PARP Inhibitors Differentially Regulate Immune Responses in Distinct Genetic Backgrounds of High-Grade Serous Tubo-Ovarian Carcinoma

**DOI:** 10.1158/2767-9764.CRC-25-0673

**Published:** 2025-11-10

**Authors:** Luiza Doro Pereira, Monica Wielgos-Bonvallet, Selim Misirlioglu, Alireza Khodadadi-Jamayran, Petar Jelinic, Douglas A. Levine

In the original version of this article (1), the name of the fourth author, Alireza Khodadadi-Jamayran, was incorrect. The name has been corrected in the latest online HTML and PDF versions of the article. The authors regret this error.
