# Supplementary material for: PARP Inhibitors Differentially Regulate Immune Responses in Distinct Genetic Backgrounds of High-Grade Serous Tubo-Ovarian Carcinoma
Source: Cancer Res Commun. 2025 Feb 19;5(2):339–48. doi: 10.1158/2767-9764.CRC-24-0515 (PMC11836641; doi:10.1158/2767-9764.CRC-24-0515)

Supplementary Figure 2: Comparison of genes that were elevated in CAOV3 and OVCAR3 cells after treatment with talazoparib or veliparib. Venn diagrams show the common genes induced (FDR<0.1) in **(A)** talazoparib-treated cells (versus DMSO controls) and **(B)** veliparib-treated (versus DMSO controls). Results shown are from three independent experiments.

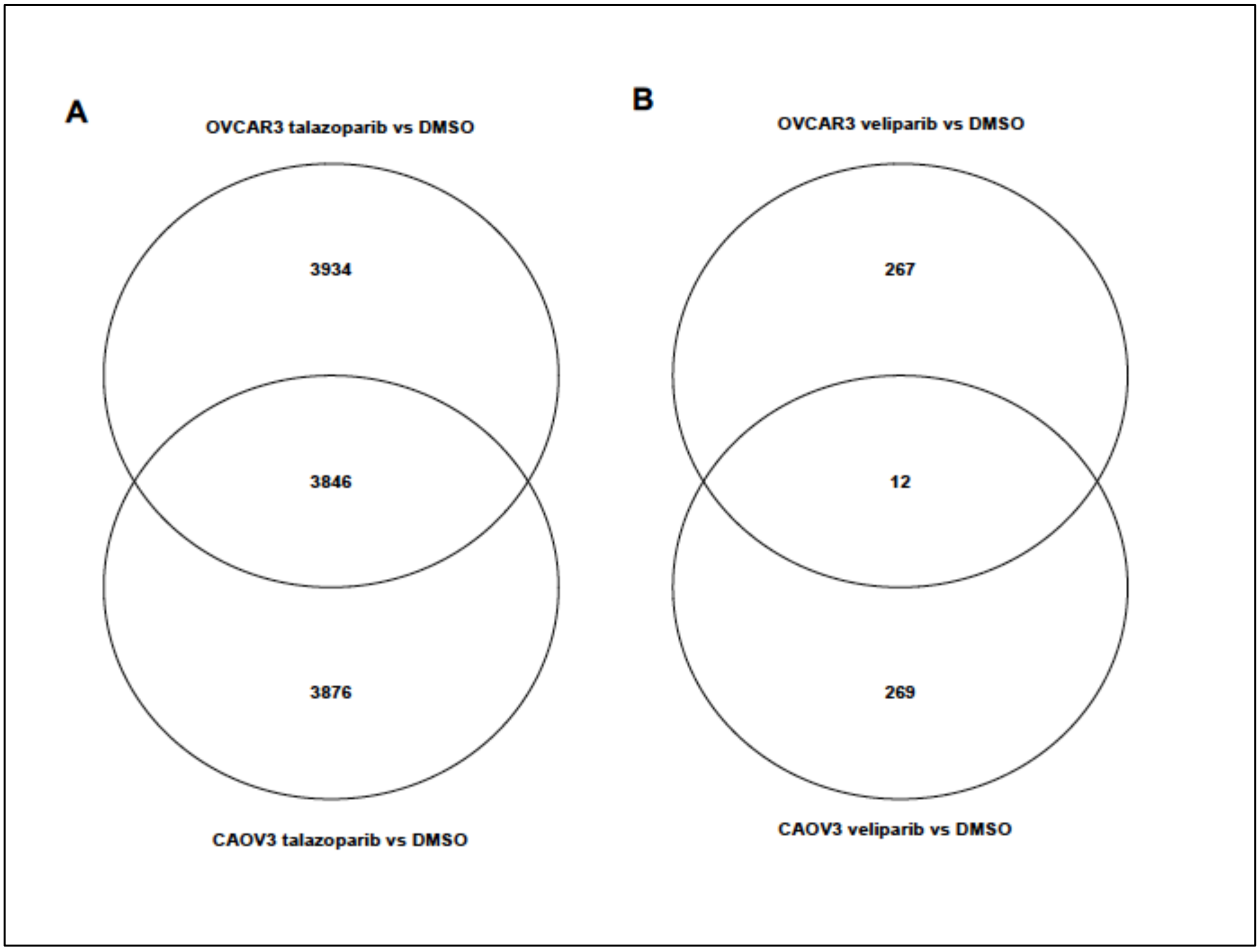

Supplement: Figure S2 — Supplementary Figure 2 shows Venn diagrams of elevated genes in drug-treated cells. [file crc-24-0515_figure_s2_suppsf2.pdf]
