# Supplementary material for: PARP Inhibitors Differentially Regulate Immune Responses in Distinct Genetic Backgrounds of High-Grade Serous Tubo-Ovarian Carcinoma
Source: Cancer Res Commun. 2025 Feb 19;5(2):339–48. doi: 10.1158/2767-9764.CRC-24-0515 (PMC11836641; doi:10.1158/2767-9764.CRC-24-0515)

**Supplementary Figure 3: Niraparib and talazoparib significantly increased *CXCL10* gene and protein expression levels compared to the three other PARP inhibitors, in a homologous recombination deficient HGSC cell line. (A,B)** OVCAR3 cells were treated with DMSO or 10 μM of veliparib, rucaparib, olaparib, niraparib, and talazoparib. Cell pellets and supernatants from both cell lines were collected 48 hours after drug treatment. **(A)** Total RNA was extracted from cells pellets that were collected 48 hours after drug treatment. Two μg of isolated RNA was reverse transcribed to cDNA and then analyzed by qRT-PCR for *CXCL10* expression. *GAPDH* was used as a housekeeping gene. **(B)** Supernatants were tested for *CXCL10 (*IP-10) protein levels using an ELISA assay. A five-parameter logistic curve fit was used to analyze these results. Means are representative of three independent experiments performed in triplicate in all qRT-PCR and ELISA experiments. T-tests were performed between DMSO and the individual PARPi treated groups. *$P$ <0.05 and **$P$ <0.01.

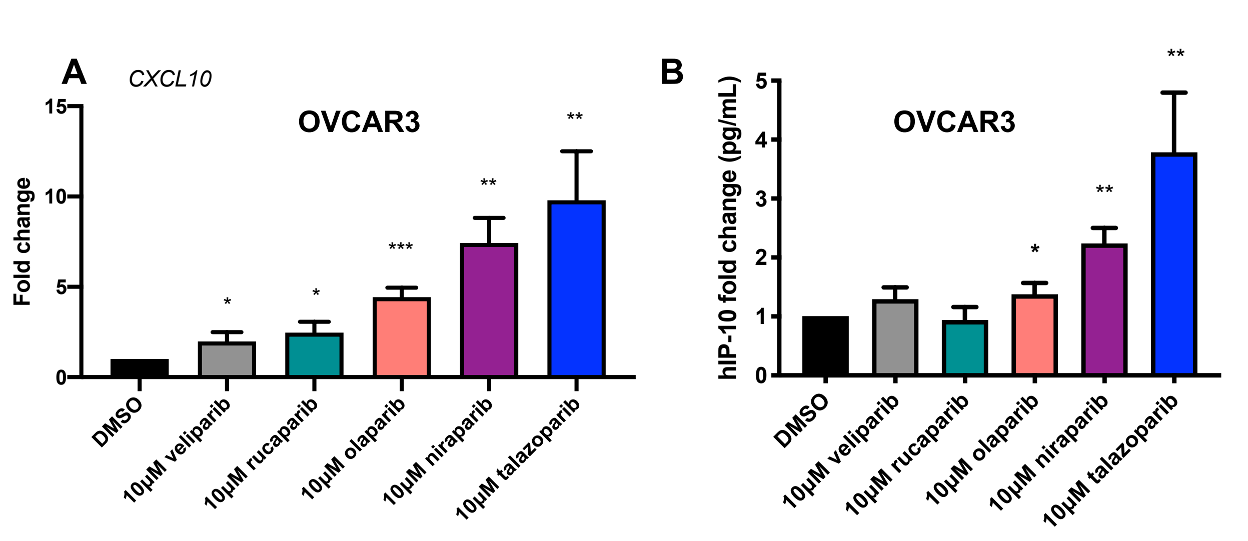

Supplement: Figure S3 — Supplementary Figure 3 shows CXCL10 gene and protein expression levels in drug-treated cell lines. [file crc-24-0515_figure_s3_suppsf3.pdf]
