# Supplementary material for: PARP Inhibitors Differentially Regulate Immune Responses in Distinct Genetic Backgrounds of High-Grade Serous Tubo-Ovarian Carcinoma
Source: Cancer Res Commun. 2025 Feb 19;5(2):339–48. doi: 10.1158/2767-9764.CRC-24-0515 (PMC11836641; doi:10.1158/2767-9764.CRC-24-0515)

Supplementary Figure 4: Olaparib and talazoparib significantly increased IP-10 (*CXCL10)* expression levels in both *BRCA1* methylated and mutated HGSC cell lines. OVCAR8 and COV362 cells were treated with a vehicle control (DMSO) or 10 μM veliparib, 10 μM olaparib, 2 μM talazoparib for 48 hours. Cell pellets and supernatants were collected 48 hours after drug treatment in the OVCAR8 cell line. Only the supernatants were collected 48 hours after drug treatment in the COV362 cell line. **(A)** Two μg of total RNA extracted from cells pellets was reverse transcribed to cDNA and then analyzed by qRT-PCR for *CXCL10* and *GAPDH* expression. **(B,C)** Supernatants were tested for *CXCL10 (*IP-10) protein levels using an ELISA assay. A five-parameter logistic curve fit was used to analyze these results. *P<0.05, **P <0.01, ***P <0.001, and ****P <0.0001.

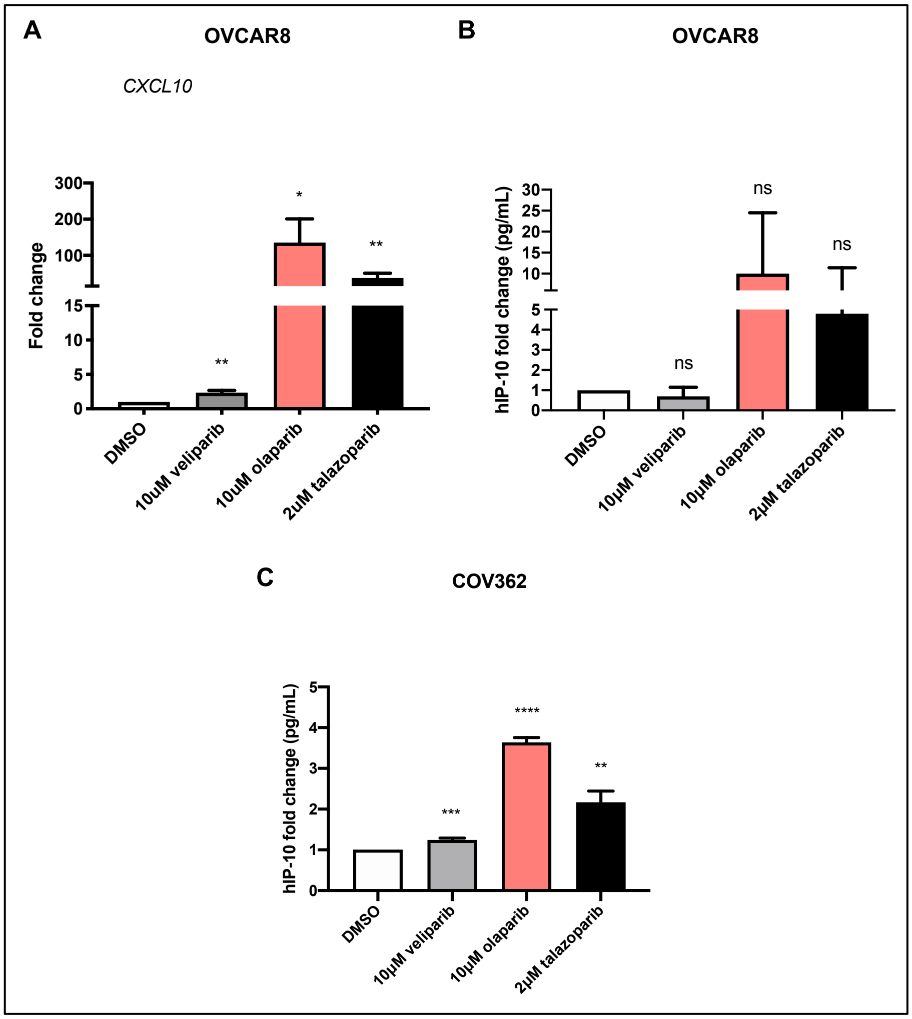

Supplement: Figure S4 — Supplementary Figure 4 shows IP-10 (CXCL10) expression levels in drug-treated cell lines. [file crc-24-0515_figure_s4_suppsf4.pdf]
