# Supplementary material for: PARP Inhibitors Differentially Regulate Immune Responses in Distinct Genetic Backgrounds of High-Grade Serous Tubo-Ovarian Carcinoma
Source: Cancer Res Commun. 2025 Feb 19;5(2):339–48. doi: 10.1158/2767-9764.CRC-24-0515 (PMC11836641; doi:10.1158/2767-9764.CRC-24-0515)
Supplement: Table S1 — Supplementary Table 1 shows enriched gene sets upregulated in CAOV3 cells treated with talazoparib. [file crc-24-0515_table_s1_suppst1.docx]

**Supplementary Table 1: Top 12 enriched ‘Hallmark’ gene sets upregulated in CAOV3 cells treated with talazoparib compared to DMSO-control cells.** Gene set enrichment analysis (GSEA) of Hallmark Pathways from rank-ordered RNA-seq data.

| ***NAME*** | ***ES*** | ***NES*** | ***NOM p-val*** | ***FDR q-val*** | ***FWER p-val*** |
| --- | --- | --- | --- | --- | --- |
| HALLMARK_TNFA_SIGNALING_VIA_NFKB | 0.5413928 | 2.3457725 | 0 | 0 | 0 |
| HALLMARK_MYC_TARGETS_V2 | 0.52551425 | 1.8817127 | 0 | 0 | 0 |
| HALLMARK_INTERFERON_GAMMA_RESPONSE | 0.37863004 | 1.6425554 | 0 | 0.016743114 | 0.036 |
| HALLMARK_INTERFERON_ALPHA_RESPONSE | 0.42249665 | 1.6338897 | 0 | 0.013585027 | 0.039 |
| HALLMARK_INFLAMMATORY_RESPONSE | 0.37766442 | 1.6155268 | 0 | 0.012389598 | 0.044 |
| HALLMARK_P53_PATHWAY | 0.3418383 | 1.4878597 | 0 | 0.04555277 | 0.177 |
| HALLMARK_KRAS_SIGNALING_UP | 0.3415194 | 1.4538772 | 0.004385965 | 0.054626424 | 0.245 |
| HALLMARK_APOPTOSIS | 0.34532264 | 1.435408 | 0.004301075 | 0.05596564 | 0.282 |
| HALLMARK_IL2_STAT5_SIGNALING | 0.33002913 | 1.4161563 | 0.006593407 | 0.061376393 | 0.336 |
| HALLMARK_IL6_JAK_STAT3_SIGNALING | 0.37574056 | 1.3994093 | 0.041666668 | 0.062919125 | 0.374 |
| HALLMARK_UV_RESPONSE_UP | 0.30684006 | 1.2879884 | 0.04017857 | 0.15475653 | 0.739 |
| HALLMARK__EPITHELIAL_MESENCHYMAL_TRANSITION | 0.28831384 | 1.2380785 | 0.05463183 | 0.21914591 | 0.857 |
