# Supplementary material for: PARP Inhibitors Differentially Regulate Immune Responses in Distinct Genetic Backgrounds of High-Grade Serous Tubo-Ovarian Carcinoma
Source: Cancer Res Commun. 2025 Feb 19;5(2):339–48. doi: 10.1158/2767-9764.CRC-24-0515 (PMC11836641; doi:10.1158/2767-9764.CRC-24-0515)
Supplement: Table S2 — Supplementary Table 2 shows enriched gene sets upregulated in OVCAR3 cells treated with talazoparib. [file crc-24-0515_table_s2_suppst2.docx]

**Supplementary Table 2: Top 12 enriched ‘Hallmark’ gene sets upregulated in OVCAR3 cells treated with talazoparib compared to DMSO-control cells.** Gene set enrichment analysis (GSEA) of Hallmark Pathways from rank-ordered RNA-seq data.

| ***NAME*** | ***ES*** | ***NES*** | ***NOM p-val*** | ***FDR q-val*** | ***FWER p-val*** |
| --- | --- | --- | --- | --- | --- |
| HALLMARK_INFLAMMATORY_RESPONSE | 0.6447164 | 2.3618271 | 0 | 0 | 0 |
| HALLMARK_IL6_JAK_STAT3_SIGNALING | 0.60814714 | 2.0639148 | 0 | 0 | 0 |
| HALLMARK_EPITHELIAL_MESENCHYMAL_TRANSITION | 0.53381103 | 1.941455 | 0 | 0 | 0 |
| HALLMARK_COAGULATION | 0.5481957 | 1.9276254 | 0 | 0 | 0 |
| HALLMARK_ALLOGRAFT_REJECTION | 0.52023834 | 1.8841411 | 0 | 0 | 0 |
| HALLMARK_KRAS_SIGNALING_UP | 0.519847 | 1.8763989 | 0 | 0 | 0 |
| HALLMARK_COMPLEMENT | 0.49450785 | 1.8086128 | 0 | 3.83E-04 | 0.003 |
| HALLMARK_KRAS_SIGNALING_DN | 0.49725506 | 1.7970705 | 0 | 4.48E-04 | 0.004 |
| HALLMARK_TNFA_SIGNALING_VIA_NFKB | 0.48495913 | 1.7893213 | 0 | 3.99E-04 | 0.004 |
| HALLMARK_INTERFERON_GAMMA_RESPONSE | 0.46460348 | 1.7091279 | 0 | 0.001651777 | 0.019 |
| HALLMARK_INTERFERON_ALPHA_RESPONSE | 0.48397854 | 1.6548096 | 0 | 0.002944037 | 0.037 |
| HALLMARK_MYOGENESIS | 0.44624183 | 1.6369109 | 0 | 0.00313787 | 0.043 |
