# Supplementary material for: PARP Inhibitors Differentially Regulate Immune Responses in Distinct Genetic Backgrounds of High-Grade Serous Tubo-Ovarian Carcinoma
Source: Cancer Res Commun. 2025 Feb 19;5(2):339–48. doi: 10.1158/2767-9764.CRC-24-0515 (PMC11836641; doi:10.1158/2767-9764.CRC-24-0515)
Supplement: Table S3 — Supplementary Table 3 shows enriched gene sets upregulated in OVCAR3 cells treated with veliparib . [file crc-24-0515_table_s3_suppst3.docx]

**Supplementary Table 3: Top 12 enriched ‘Hallmark’ gene sets upregulated in OVCAR3 cells treated with veliparib compared to DMSO-control cells.** Gene set enrichment analysis (GSEA) of Hallmark Pathways from rank-ordered RNA-seq data.

| ***NAME*** | ***ES*** | ***NES*** | ***NOM p-val*** | ***FDR q-val*** | ***FWER p-val*** |
| --- | --- | --- | --- | --- | --- |
| HALLMARK_INFLAMMATORY_RESPONSE | 0.52420795 | 1.7057835 | 0 | 0.012669235 | 0.012 |
| HALLMARK_ALLOGRAFT_REJECTION | 0.4971636 | 1.6109064 | 0 | 0.02400498 | 0.043 |
| HALLMARK_EPITHELIAL_MESENCHYMAL_TRANSITION | 0.48549008 | 1.5860147 | 0 | 0.022334615 | 0.059 |
| HALLMARK_TNFA_SIGNALING_VIA_NFKB | 0.47457427 | 1.5664616 | 0 | 0.019717658 | 0.068 |
| HALLMARK_INTERFERON_GAMMA_RESPONSE | 0.42415893 | 1.3819163 | 0.008321775 | 0.12880415 | 0.442 |
| HALLMARK_APOPTOSIS | 0.41065487 | 1.3399174 | 0.017366135 | 0.1734839 | 0.617 |
| HALLMARK_INTERFERON_ALPHA_RESPONSE | 0.43576697 | 1.3282878 | 0.03271028 | 0.16674998 | 0.659 |
| HALLMARK_MYOGENESIS | 0.40101117 | 1.3049023 | 0.029453015 | 0.18679318 | 0.735 |
| HALLMARK_UV_RESPONSE_UP | 0.40244108 | 1.2901583 | 0.029957203 | 0.19594479 | 0.79 |
| HALLMARK_APICAL_JUNCTION | 0.37636817 | 1.2251154 | 0.052249637 | 0.3241535 | 0.943 |
| HALLMARK_KRAS_SIGNALING_DN | 0.37423342 | 1.205297 | 0.0918221 | 0.35593522 | 0.964 |
| HALLMARK_COMPLEMENT | 0.36541745 | 1.1913345 | 0.09052334 | 0.36857885 | 0.98 |
