# Supplementary material for: PARP Inhibitors Differentially Regulate Immune Responses in Distinct Genetic Backgrounds of High-Grade Serous Tubo-Ovarian Carcinoma
Source: Cancer Res Commun. 2025 Feb 19;5(2):339–48. doi: 10.1158/2767-9764.CRC-24-0515 (PMC11836641; doi:10.1158/2767-9764.CRC-24-0515)
Supplement: Table S4 — Supplementary Table 4 shows enriched gene sets upregulated in CAOV3 cells treated with veliparib . [file crc-24-0515_table_s4_suppst4.docx]

**Supplementary Table 4: Top 12 enriched ‘Hallmark’ gene sets upregulated in CAOV3 cells treated with veliparib compared to DMSO-control cells.** Gene set enrichment analysis (GSEA) of Hallmark Pathways from rank-ordered RNA-seq data.

| ***NAME*** | ***ES*** | ***NES*** | ***NOM p-val*** | ***FDR q-val*** | ***FWER p-val*** |
| --- | --- | --- | --- | --- | --- |
| HALLMARK_MYC_TARGETS_V2 | 0.29477957 | 0.86653876 | 0.76938367 | 1 | 1 |
| HALLMARK_OXIDATIVE_PHOSPHORYLATION | 0.24422598 | 0.84664124 | 0.9337607 | 1 | 1 |
| HALLMARK_COAGULATION | 0.22328952 | 0.72748196 | 0.9939148 | 1 | 1 |
| HALLMARK_KRAS_SIGNALING_DN | 0.21233523 | 0.7262426 | 1 | 1 | 1 |
| HALLMARK_EPITHELIAL_MESENCHYMAL_TRANSITION | 0.20293881 | 0.70434636 | 1 | 1 | 1 |
| HALLMARK_XENOBIOTIC_METABOLISM | 0.2043688 | 0.7019639 | 1 | 1 | 1 |
| HALLMARK_TGF_BETA_SIGNALING | 0.23936291 | 0.6915535 | 0.97987926 | 1 | 1 |
| HALLMARK_APICAL_JUNCTION | 0.19484179 | 0.6744729 | 1 | 1 | 1 |
| HALLMARK_PROTEIN_SECRETION | 0.20788962 | 0.6667543 | 0.9980392 | 1 | 1 |
| HALLMARK_FATTY_ACID_METABOLISM | 0.19615887 | 0.65712744 | 1 | 1 | 1 |
| HALLMARK_SPERMATOGENESIS | 0.19711913 | 0.63933134 | 1 | 1 | 1 |
| HALLMARK_UNFOLDED_PROTEIN_RESPONSE | 0.1908373 | 0.6191104 | 1 | 1 | 1 |
