# Supplementary material for: PARP Inhibitors Differentially Regulate Immune Responses in Distinct Genetic Backgrounds of High-Grade Serous Tubo-Ovarian Carcinoma
Source: Cancer Res Commun. 2025 Feb 19;5(2):339–48. doi: 10.1158/2767-9764.CRC-24-0515 (PMC11836641; doi:10.1158/2767-9764.CRC-24-0515)

Supplementary Figure 1: Distinct clustering is observed in talazoparib-treated cells compared to control and veliparib-treated cells. The hierarchical clustering heatmap is based on Euclidean distances calculated from normalized RNA sequence count data for CAOV3 and OVCAR3 cells treated with DMSO, 10μM veliparib, or 2μM talazoparib for 48 hours. Results shown are from three independent experiments.

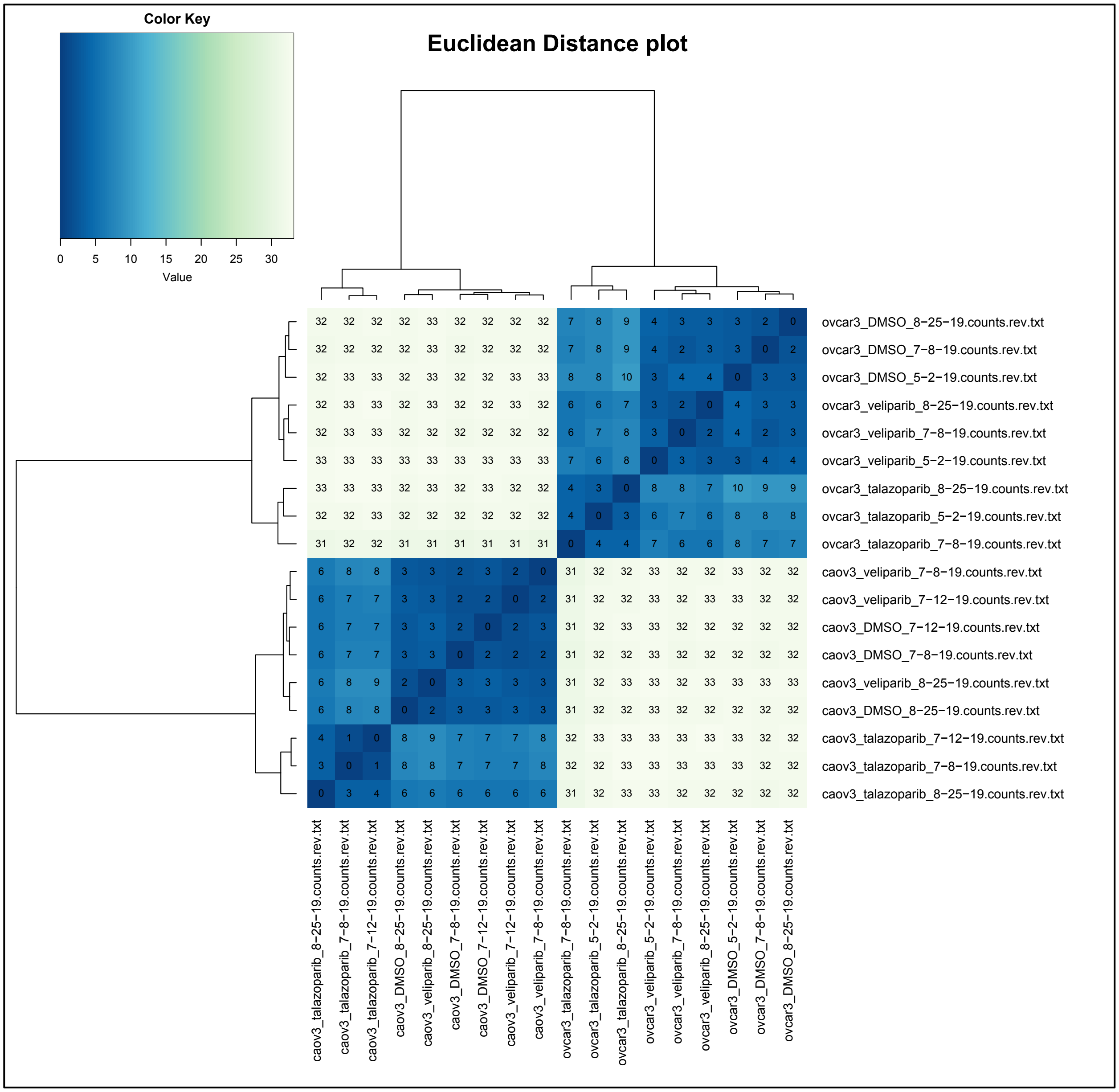

Supplement: Figure S1 — Supplementary Figure 1 shows clustering of drug-treated cells compared to controls. [file crc-24-0515_figure_s1_suppsf1.pdf]
